# Investigating the Relationship Between Serum Uric Acid and Dyslipidemia in Young Adults in Bangladesh

**DOI:** 10.1002/edm2.70063

**Published:** 2025-05-27

**Authors:** Abu Taher, Aporajita Das Trisha, Shamim Ahmed, Jannat Begum, Falguni Sinha, Nusrat Zaman Sarna, Nurshad Ali

**Affiliations:** ^1^ Department of Biochemistry and Molecular Biology Shahjalal University of Science and Technology Sylhet Bangladesh

**Keywords:** Bangladesh, cardiovascular disease, dyslipidemia, serum uric acid, young adults

## Abstract

**Objectives:**

While some reports exist on the relationship between serum uric acid (SUA) and dyslipidemia in adults, there is limited information available regarding young adults. This study aimed to evaluate the relationship between SUA and dyslipidemia, as well as its components, among young adults in Bangladesh.

**Methods:**

This study consisted of 458 participants (281 male and 177 female) aged between 18 and 30 years. The levels of SUA, fasting blood glucose and lipid profile (TG, TC, HDL‐C and LDL‐C) were measured using standard colorimetric methods. Bivariate logistic regression modelling was used to examine the relationship between SUA and dyslipidemia and its components.

**Results:**

The overall prevalence of hyperuricemia was 24% with 27.6% in males and 18.6% in females. Males had a higher mean SUA level (6.6 ± 1.5 mg/dL) than females (5.3 ± 1.2 mg/dL) (*p* < 0.001). The prevalence of dyslipidemia was 74.2% with 83.2% in male and 59.8% in female subjects. The prevalence of hypertriglyceridemia, hypercholesterolemia, high LDL‐C and low HDL‐C was 30.1%, 26.2%, 28.8% and 64.8%, respectively. There was an increasing trend in the level and prevalence of elevated lipid profile markers across the SUA quartiles (*p* < 0.001). SUA level showed a positive correlation with TG, TC and LDL‐C and a negative correlation with HDL (*p* < 0.001). In regression analysis, a significant association was found between SUA and dyslipidemia in all participants as well as in the male–female groups separately (at least *p* < 0.05). Furthermore, a significant association (*p* < 0.001) was found between SUA and individual lipid components in the regression models.

**Conclusion:**

Dyslipidemia and its components were more prevalent in individuals with hyperuricemia than in those without. This study identified a significant association between SUA and dyslipidemia in young adults in Bangladesh. Further research is needed to explore the mechanisms behind this association.

## Graphical abstract

A significant positive correlation was found between SUA and serum level of TG, TC and LDL‐C. Conversely, a significant negative correlation was observed between SUA and serum level of HDL‐C. In regression models, SUA showed an independent association with dyslipidemia and its individual components.

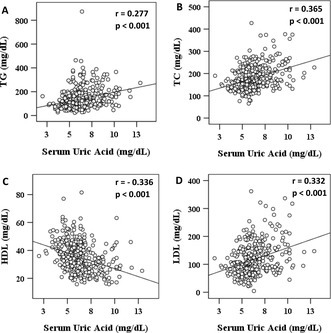

## 1 Introduction

Uric acid is the final breakdown product of purine nucleotides. Approximately 70% of serum uric acid (SUA) is excreted through the kidneys, with a small portion eliminated via biliary and intestinal secretion in the human body [1]. Disruptions in SUA metabolism and reduced kidney excretion can lead to hyperuricemia [2]. Epidemiological studies indicate that hyperuricemia is becoming more common in both developing and developed countries [3]. Hyperuricemia is associated with various health conditions, including diabetes, hypertension, cardiovascular disease (CVD), gout, metabolic syndrome, liver dysfunction and renal dysfunction [4, 5, 6, 7, 8, 9, 10].

Dyslipidemia refers to an abnormal condition characterised by irregular levels of lipids in the blood, specifically elevated total cholesterol (TC), triglycerides (TG) and low‐density lipoprotein cholesterol (LDL‐C), along with reduced levels of high‐density lipoprotein cholesterol (HDL‐C). Dyslipidemia is closely linked to obesity, type 2 diabetes mellitus, metabolic syndrome, and nonalcoholic fatty liver disease, and these conditions can lead to significant public health challenges globally [11, 12, 13]. Over recent decades, the prevalence of dyslipidemia has increased in many developing countries due to lifestyle changes associated with economic development [14]. Among the various forms of dyslipidemia, hypercholesterolemia is the most common and is associated with a higher risk of CVD. A recent review indicated a high prevalence of CVD in the Bangladeshi population [15]. In fact, elevated LDL‐C levels were identified as a leading cause of death globally in 2019 [16]. Over the past three decades, dyslipidemia has become increasingly prevalent and is now recognised as a significant global health issue affecting both developed and developing countries [14, 17].

The connection between SUA levels and lipid profiles has drawn significant interest over time. However, the relationship between SUA levels and dyslipidemia, as well as its individual components, is not yet fully understood. Some studies have shown a significant correlation between SUA levels and TG, but not with HDL‐C [18]. It also remains unclear whether the association between SUA levels and dyslipidemia is consistent across different genders, which should be further examined. A recent cohort study identified a strong positive association between SUA levels and dyslipidemia in male participants but not in females [19]. Therefore, additional research is warranted to explore the relationship between SUA levels and dyslipidemia more comprehensively. Moreover, the exact role of uric acid in these conditions continues to be debated, as it often coexists with other risk factors, such as diet and obesity. There is ongoing discussion about whether SUA is a causative risk factor or merely a coexisting marker of these pathological processes.

While the risk factors for metabolic syndrome and CVD are being extensively studied, few studies have examined the association between SUA and dyslipidemia in the adult population [20, 21, 22], and there is limited information available regarding young adults. Young adults play a vital role in society by significantly contributing to the workforce and ensuring the well‐being of future generations. Therefore, identifying disease prevalence and associated risk factors at an early stage may help reduce the disease burden later in life. Consequently, the high prevalence of hyperuricemia, dyslipidemia, or both in this young age group is of concern. In this study, we aimed to investigate the association between SUA levels and dyslipidemia, along with its components in young adults in Bangladesh. Additionally, we sought to explore the age‐ and gender‐specific associations of SUA levels with dyslipidemia among young adults.

## 2 Methods

### 2.1 Study Design and Participant Recruitment

**FIGURE 1:**
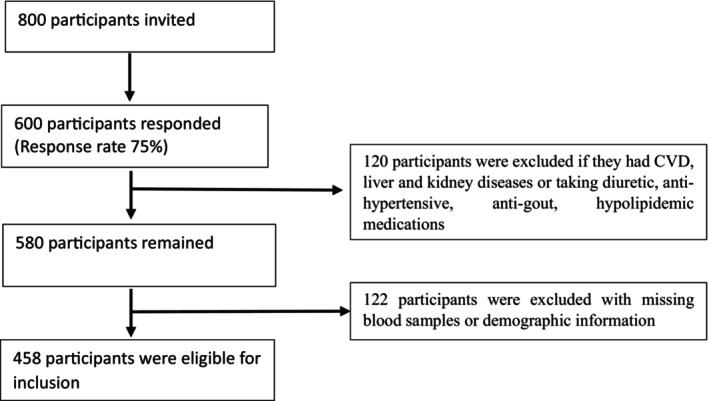
Flow chart of sampling.

This study was conducted in a cross‐sectional manner, involving 458 young adult participants (281 males and 177 females) recruited between October 2022 and March 2024 from the Sylhet region of Bangladesh. The participants were enrolled from university and college level students, as well as age and sex‐matched individuals from urban, suburban and rural areas in Sylhet. A simple random sampling technique was used to determine the study population. We invited 800 participants, 600 of whom responded and 458 were eligible to participate in the study (Figure 1). Participant inclusion criteria were (i) willingness to take part; (ii) both sexes; and (iii) age between 18 and 30 years. Participant exclusion criteria were (i) participants with CVD, liver and kidney diseases; (ii) participants taking the medication of diuretic, cytotoxic drugs, anti‐hypertensive, anti‐diabetic, anti‐gout, hypolipidaemic and alcoholics; (iv) pregnant and nursing mothers; (v) individuals with absent blood samples or incomplete questionnaire form. All biochemical analyses were carried out at the Department of Biochemistry and Molecular Biology in Shahjalal University of Science and Technology (SUST), Sylhet, Bangladesh. The study protocol was approved by the Ethics Review Committee at the Department of Biochemistry and Molecular Biology, School of Life Sciences, SUST (Reference no. 02/BMB/2019). Participants were informed about the study aim and provided written consent prior to participating. All steps in the study protocol were conducted in accordance with institutional guiding principles and regulations.

### 2.2 General Data Collection

The demographic and lifestyle data were collected using a standard questionnaire described elsewhere [6, 23, 24, 25, 26]. Body weight (BW) was measured using a calibrated modern digital weighing machine (Beurer 700, Germany) to the nearest 0.1 kg, and body height (BH) was recorded to the nearest 0.1 cm using the height measuring tape. Body Mass Index (BMI) was calculated as weight in kilograms divided by height in meters squared. Waist circumference (WC) was measured midway between the lowest rib and the iliac crest, while hip circumference (HC) was measured at the largest buttocks circumference, both to the nearest 0.5 cm using an anthropometric tape. Blood pressure readings were taken using the Omron M10 device from Omron Corporation in Tokyo, Japan, following a 10‐min relaxation period. Three consecutive readings were recorded within a 5‐min interval. The average of the second and third measurements was used to determine the systolic and diastolic blood pressures (SBP and DBP, respectively). The accuracy of anthropometric measurements was ensured by the presence of investigators.

### 2.3 Sample Collection and Laboratory Measurements

The participants provided 5 mL of venous blood samples after fasting overnight. The blood was allowed to clot at room temperature and then centrifuged at 3000 rpm for 15 min to separate the serum. The isolated serum was stored in a −20°C refrigerator until biochemical analysis. Biochemical parameters, including SUA, fasting blood glucose (FBG), TC, TG, HDL‐C and LDL‐C, were measured using a colorimetric method, following the manufacturer's protocol (HUMAN Gesellschaft für Biochemica und Diagnostica mbH, Germany) with a semi‐automatic biochemistry analyser (Humalyzer 3000, Medicon Services, Germany). The accuracy of the measurements was regularly maintained through standard method calibration.

### 2.4 Diagnostic Criteria

Hyperuricemia was defined as SUA levels > 7.0 mg/dL (416.4 μmol/L) in men or > 6.0 mg/dL (356.9 μmol/L) in women [27, 28]. The SUA levels were divided into four quartiles: Q1: ≤ 5.3 mg/dL; Q2: 5.4–6.0 mg/dL; Q3: 6.1–6.8 mg/dL; and Q4: 6.9–12.9 mg/dL. Dyslipidemia was defined according to the National Cholesterol Education Program—Adult Treatment Panel III (NCEP‐ATP‐III) model guideline [29]. The isolated components of dyslipidemia included: (i) elevated TC ≥ 200 mg/dL; (ii) elevated TG ≥ 150 mg/dL; (iii) low HDL‐C < 40 mg/dL; and (iv) elevated LDL‐C ≥ 130 mg/dL. Participants with at least one of these components, or a combination of them, were identified as having dyslipidemia. Physical activity was defined based on the Global Physical Activity Questionnaire (GPAQ) developed by the World Health Organization (WHO) [30]. Physical activity was classified into three categories: low, moderate and vigorous depending on the level of exertion involved. Examples of low activity include office work and light housework. Moderate activity encompasses general walking, swimming and light cleaning. Vigorous activity includes tasks such as lifting, carrying, jogging and participating in sports [31].

### 2.5 Statistical Analysis

Data analysis was carried out using IBM SPSS statistics software version 25 (SPSS Inc., Chicago, Illinois, USA). Descriptive data were presented as mean ± standard deviation and SUA as quartile ranges. Categorical data were presented as percentages (%) using the chi‐squared test. Differences between gender groups were assessed by an independent sample *t*‐test (two‐tailed). One‐way ANOVA was used to compare the baseline parameters in the SUA quartiles. Pearson's correlation was used to determine the correlation between SUA and dyslipidemia. The association between SUA and dyslipidemia and its components was assessed by binary logistic regression models. Dyslipidemia was classified as present (yes) or absent (no). In the regression analysis, dyslipidemia (yes or no) and its components elevated (yes or no) were treated as the dependent variable, while SUA was regarded as the independent variable. Three models were adjusted in the regression analysis: model 1: age, gender and BMI; model 2: parameters in model 1 and WC and HC; and model 3: parameters included in model 2 and smoking and physical activity. Subsequently, this association was further verified by analysing SUA quartile groups through multinomial logistic regression analysis, with adjustments made for several confounding factors. A *p*‐value of < 0.05 was considered statistically significant for all statistical analyses.

## 3 Results

### 3.1 Characteristics of the Study Participants

The baseline characteristics of the study subjects based on gender are presented in Table 1. Out of 458 participants, 281 (61.4%) were males and 177 (38.6%) were females. The age range of the participants was between 18 and 30 years (mean 23.9 ± 2.6 years). The average BMI for all study participants was 23.2 ± 3.6 kg/m^2^ with a significant difference between male (23.8 ± 3.6 kg/m^2^) and female (22.3 ± 3.5 kg/m^2^) participants (*p* < 0.001). There were significant differences in SUA and lipid profile components (TG, TC, HDL‐C and LDL‐C) between male and female groups (at least *p* < 0.05 for all cases). The overall prevalence of hyperuricemia was 24% with a higher prevalence in males (27.4%) than in females (18.6%) subjects (*p* < 0.05). There was a significant difference in smoking and physical activity between the gender groups (*p* < 0.001). When the study participants were further characterised based on SUA levels, a noticeable increasing trend (except for HDL‐C) was observed in the levels of most of the biochemical parameters across the SUA quartiles (Table 2). A significant difference was found for BMI, WC, HC, SBP, DBP, TG, TC, HDL‐C, LDL‐C, smoking status and physical activity across the SUA quartiles.

**TABLE 1:** Study population's baseline characteristics.

Variable	Total	Male	Female	*p*
*N*	458	281	177	—
Age (year)	23.9 ± 2.6	24.4 ± 2.7	23 ± 2	< 0.001
BMI (kg/m^2^)	23.2 ± 3.6	23.8 ± 3.6	22.3 ± 3.5	< 0.001
WC (cm)	82.9 ± 9.8	85.2 ± 9.3	79.3 ± 9.6	< 0.001
HC (cm)	94.8 ± 8.3	95.6 ± 8.3	93.6 ± 8.1	0.012
SBP (mmHg)	119.1 ± 12	123.4 ± 10.9	112.3 ± 10.5	< 0.001
DBP (mmHg)	73.8 ± 10.1	76 ± 9.7	70.3 ± 9.9	< 0.001
FBG (mmol/L)	5.1 ± 1.1	5.1 ± 1.1	5.1 ± 1.2	0.992
SUA (mg/dL)	6.1 ± 1.5	6.6 ± 1.5	5.3 ± 1.2	< 0.001
TG (mg/dL)	132.9 ± 77.3	153.2 ± 85.8	100.9 ± 46.3	< 0.001
TC (mg/dL)	176.2 ± 51.4	182.7 ± 50.2	166.1 ± 51.8	0.001
HDL‐C (mg/dL)	35.8 ± 10.9	32.7 ± 9.7	40.9 ± 10.9	< 0.001
LDL‐C (mg/dL)	113.6 ± 52.7	119 ± 52.4	105 ± 52	0.003
Hyperuricemia (%)	24.0	27.4	18.6	0.033
Smoking (%)				
No	84.8	76.6	97.7	< 0.001
Yes	15.2	23.4	2.3	
Physical activity *n* (%)				
Low	20.6	17.2	26	
Moderate	72.4	72.4	72.3	< 0.001
Adequate	7	10.4	1.7	

*Note:* The results are expressed as mean ± SD or %. *p*‐values are obtained from independent sample *t*‐tests for continuous variables and chi‐squared tests for categorical variables.

**TABLE 2:** SUA quartile‐based baseline characteristics.

SUA levels (mg/dL)						
	Overall	Q1 (≤ 5.1)	Q2 (5.2–5.9)	Q3 (6.0–6.8)	Q4 (≥ 6.9)	*p*
Number	458	114	128	113	103	—
Gender (M/F)	281/177	28/86	70/58	95/18	88/15	—
Age (year)	23.9 ± 2.6	23.6 ± 2.3	23.9 ± 2.7	23.9 ± 2.8	24.1 ± 2.5	0.363
BMI (kg/m^2^)	23.2 ± 3.6	21.6 ± 3.3	23.2 ± 3.2	23.0 ± 3.5	25.3 ± 3.6	< 0.001
WC (cm)	82.9 ± 9.8	78 ± 9.9	82.7 ± 8.3	82.9 ± 8.9	88.6 ± 9.5	< 0.001
HC (cm)	94.8 ± 8.3	91.4 ± 8.7	95.2 ± 6.8	94.3 ± 7.8	98.8 ± 8.2	< 0.001
SBP (mmHg)	119.1 ± 12	115.6 ± 12.3	118.5 ± 13	119.5 ± 11	123.3 ± 10.4	< 0.001
DBP (mmHg)	73.8 ± 10.1	72.3 ± 10	72.8 ± 10.7	73.4 ± 10.6	77 ± 8.4	0.001
FBG (mmol/L)	5.1 ± 1.1	5.3 ± 1.6	5 ± 0.7	5.2 ± 1.1	5.1 ± 1.0	0.163
SUA (mg/dL)	6.1 ± 1.5	4.4 ± 0.6	5.5 ± 0.2	6.3 ± 0.2	8.4 ± 1.2	< 0.001
TG (mg/dL)	132.9 ± 77.3	101.2 ± 48.5	128.2 ± 76.3	136.4 ± 83.7	168.9 ± 81.3	< 0.001
TC (mg/dL)	176.2 ± 51.4	157.4 ± 39.7	168.9 ± 49.2	175.7 ± 47.7	205.8 ± 57.1	< 0.001
HDL‐C (mg/dL)	35.8 ± 10.9	41.9 ± 11.7	36.2 ± 9.4	34.8 ± 10.7	29.9 ± 8.1	< 0.001
LDL‐C (mg/dL)	113.6 ± 52.7	95.2 ± 38.8	107 ± 49.7	112.9 ± 48.9	142 ± 61.8	< 0.001
Smoking (%)						
No	84.5	95.6	81.3	77	84.5	0.001
Yes	15.5	4.4	18.8	23	15.5	
Physical activity *n* (%)						
Low	20.6	21.1	20.5	17.9	23.3	
Moderate	72.4	76.3	71.7	70.5	70.9	0.242
Adequate	7	2.6	7.9	11.6	5.8	

*Note:* The data are expressed as mean ± SD or %. *p*‐values are obtained from One‐way ANOVA for continuous variables and chi‐squared test for categorical values.

### 3.2 Prevalence of Dyslipidemia in Different Groups

**FIGURE 2:**
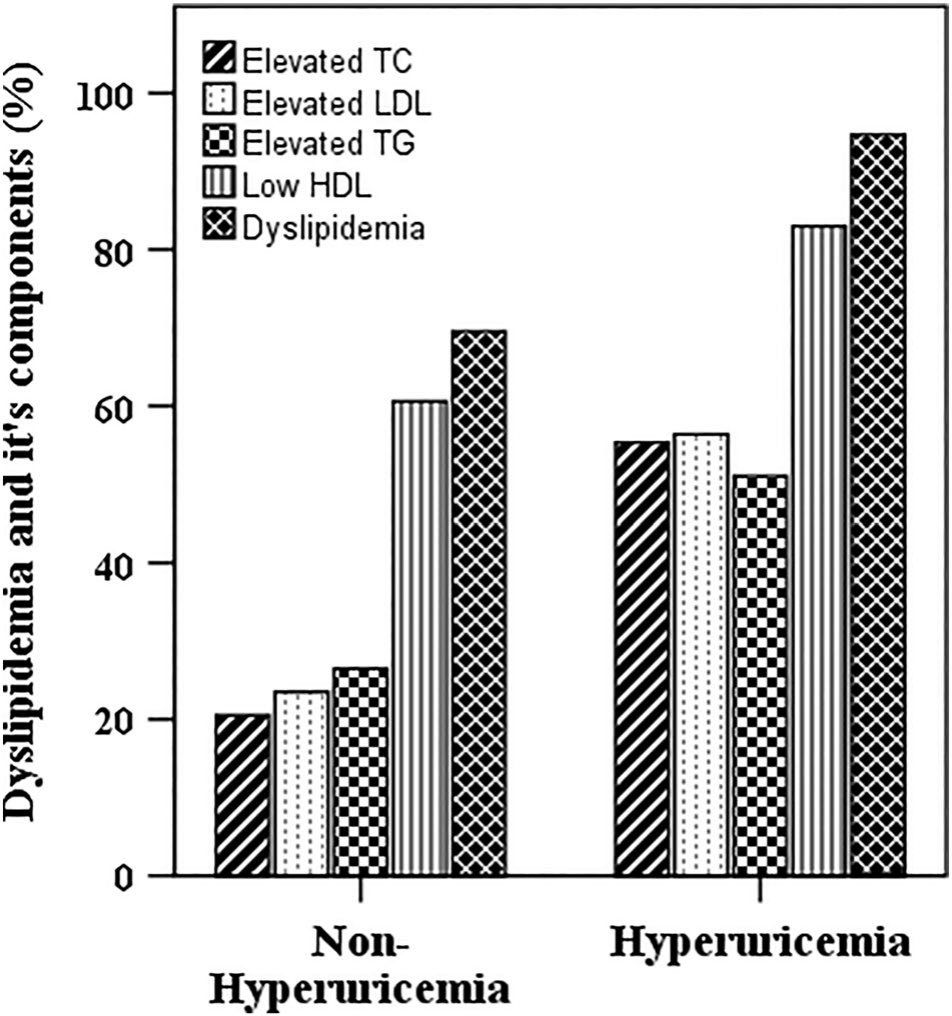
Prevalence of dyslipidemia and its components in the hyperuricemia and non‐hyperuricemia group. *p* < 0.001 when the prevalence of dyslipidemia and its individual components is compared between the groups. *p*‐values are obtained from the Chi‐squared test.

Overall dyslipidemia prevalence was 74.2% with 83.2% in male and 59.8% in female subjects (Table 3). The prevalence of isolated TG, TC, low HDL‐C and LDL‐C was 30.1%, 26.2%, 64.8% and 28.8%, respectively. The prevalence of overall dyslipidemia, hypertriglyceridemia, high LDL‐C and low HDL‐C was significantly higher in males than in the female subjects (at least *p* < 0.05 for all cases). Hyperuricemic individuals tend to have a higher level of general and isolated dyslipidemia compared to normal study subjects (*p* < 0.001) (Figure 2). When SUA levels were divided into four quartiles (Q1 to Q4), a significant increasing trend was observed in the prevalence of overall dyslipidemia and its components (except low HDL‐C, showed decreasing trend) across the SUA quartiles (*p* < 0.001) (Table 4).

**TABLE 3:** Prevalence of general and isolated dyslipidemia according to gender.

	Overall	Male	Female	*p*
Dyslipidemia (%)	74.2	83.2	59.8	< 0.001
Hypertriglyceridemia (%)	30.1	40.9	12.9	< 0.001
Hypercholesterolemia (%)	26.2	28.8	22	0.107
Low HDL‐C (%)	64.8	74	50.2	< 0.001
High LDL‐C (%)	28.8	32.7	22.5	0.020

*Note: p*‐values are obtained from Chi‐squared test. The isolated components of dyslipidemia: TC ≥ 200 mg/dL; TG ≥ 150 mg/dL; HDL‐C < 40 mg/dL and, LDL‐C ≥ 130 mg/dL. General dyslipidemia: having at least one of these components, or a combination of them [29].

**TABLE 4:** Prevalence of general isolated dyslipidemia according to the SUA quartiles.

SUA (mg/dL)						
	Overall	Q1	Q2	Q3	Q4	*p*
Dyslipidemia (%)	74.2	61.4	70.3	73.4	94.1	< 0.001
Hypertriglyceridemia (%)	30.1	14.9	25.7	31.8	50.4	< 0.001
Hypercholesterolemia (%)	26.2	16.6	20.3	20.4	50.4	< 0.001
Low HDL‐C (%)	64.8	49.1	60.1	69	83.4	< 0.001
High LDL‐C (%)	28.8	16.6	24.2	25.6	51.4	< 0.001

*Note: p*‐values are obtained from Chi‐squared test. The isolated components of dyslipidemia: TC ≥ 200 mg/dL; TG ≥ 150 mg/dL; HDL‐C < 40 mg/dL and, LDL‐C ≥ 130 mg/dL. General dyslipidemia: having at least one of these components, or a combination of them [29].

### 3.3 Relationship of SUA With Dyslipidemia and Its Components

**FIGURE 3:**
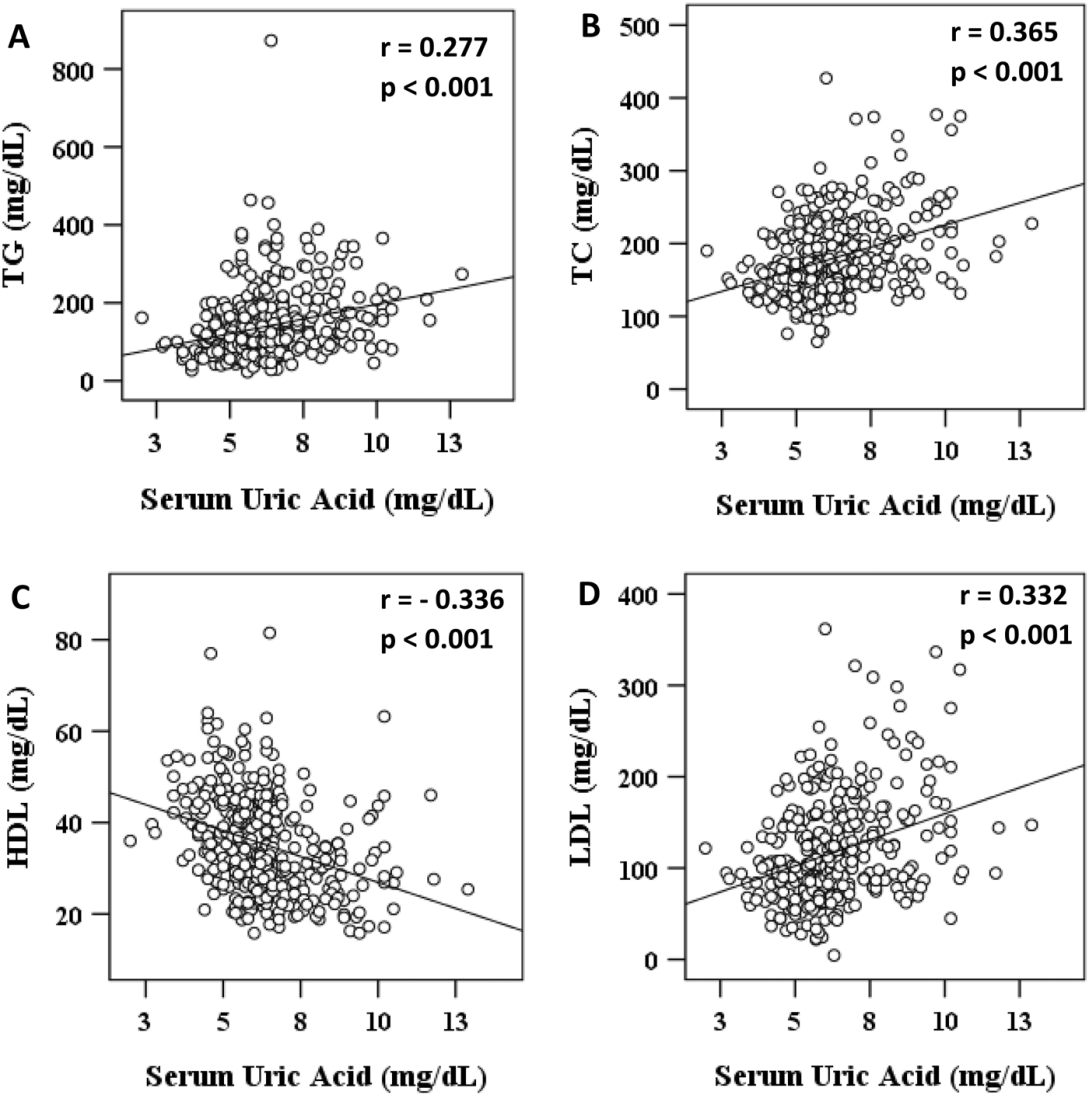
Correlation of serum uric acid with TG (A), TC (B), HDL (C) and LDL (D). The scale range in the *Y*‐axis is not the same for all figures.

A significant positive correlation (*p* < 0.001) was found between SUA levels and serum TG, TC and LDL‐C levels. Conversely, a significant negative correlation was observed between SUA levels and serum HDL‐C level (*p* < 0.001) (Figure 3). Logistic regression models were used to assess the relationship between SUA levels and dyslipidemia as well as its components.

After adjusting for age, sex and other factors in regression models, a significant association (at least *p* < 0.05) was found between SUA and dyslipidemia prevalence in all participants as well as in the male– female groups separately (Table 5). When the association between SUA and dyslipidemia components was examined, a significant association (*p* < 0.001) was observed for SUA with each lipid marker in all three models (Table 6). Furthermore, the relationship of SUA with dyslipidemia components was assessed based on SUA quartiles, and a significant association (*p* < 0.05) was found between SUA and individual lipid components in all models (Table 7).

**TABLE 5:** Logistic regression analysis to evaluate the association between SUA levels and dyslipidemia according to gender.

	*B*	SE	Wald	df	OR (95% CI)	*p*
Overall						
Model 1	0.303	0.113	7.160	1	1.35 (1.09–1.69)	< 0.01
Model 2	0.310	0.114	7.412	1	1.36 (1.09–1.71)	< 0.01
Model 3	0.287	0.117	6.031	1	1.33 (1.06–1.68)	< 0.05
Male						
Model 1	0.339	0.135	6.237	1	1.40 (1.07–1.83)	< 0.05
Model 2	0.307	0.141	4.717	1	1.36 (1.03–1.79)	< 0.05
Model 3	0.305	0.140	4.753	1	1.36 (1.03–1.79)	< 0.05
Female						
Model 1	0.462	0.201	5.318	1	1.59 (1.07–2.35)	< 0.05
Model 2	0.459	0.207	4.928	1	1.58 (1.05–2.38)	< 0.05
Model 3	0.467	0.218	4.603	1	1.60 (1.04–2.45)	< 0.05

*Note:* Binary logistic regression analysis is applied to evaluate between SUA and dyslipidemia with its each component. Dependent variable is dyslipidemia defined as yes (1) or no (1), and independent variable is SUA (mg/dL). Adjusted covariates in regression model 1: age; model 2: covariates in model 1 and BMI, WC and HC; model 3: covariates in model 2 and smoking and physical activity.

Abbreviations: CI, confidence interval; OR, odds ratio; SE, standard error.

**TABLE 6:** Logistic regression analysis to evaluate the association between SUA levels and individual components of dyslipidemia.

	*B*	SE	Wald	df	OR (95% CI)	*p*
TG						
Model 1	0.016	0.003	23.329	1	1.02 (1.01–1.02)	< 0.001
Model 2	0.016	0.003	22.889	1	1.02 (1.01–1.02)	< 0.001
Model 3	0.016	0.003	21.554	1	1.02 (1.01–1.02)	< 0.001
TC						
Model 1	0.022	0.04	29.063	1	1.02 (1.01–1.03)	< 0.001
Model 2	0.021	0.04	28.510	1	1.02 (1.01–1.03)	< 0.001
Model 3	0.021	0.04	26.366	1	1.02 (1.01–1.03)	< 0.001
HDL‐C						
Model 1	−0.241	0.028	70.151	1	0.79 (0.74–0.83)	< 0.001
Model 2	−0.244	0.029	70.234	1	0.78 (0.74–0.83)	< 0.001
Model 3	−0.250	0.030	65.746	1	0.78 (0.73–0.83)	< 0.001
LDL‐C						
Model 1	0.026	0.004	37.148	1	1.03 (1.02–1.03)	< 0.001
Model 2	0.026	0.004	36.820	1	1.03 (1.02–1.04)	< 0.001
Model 3	0.026	0.004	34.728	1	1.03 (1.02–1.04)	< 0.001

*Note:* Binary logistic regression models is applied to evaluate between SUA and dyslipidemia components. Dependent variable is elevated dyslipidemia component defined as yes (1) or no (0), and independent variable is SUA (mg/dL). Adjusted covariates in regression model 1: age and gender; model 2: covariates in model 1 and BMI, WC and HC; model 3: covariates in model 2 and smoking and physical activity.

Abbreviations: CI, confidence interval; OR, odds ratio; SE, standard error.

**TABLE 7:** Association of SUA with dyslipidemia components according to SUA quartiles.

	OR (95% CI)				*p* for trend
	Q1 (< 5.2)	Q2 (5.2–6.2)	Q3 (6.3–7.4)	Q4 (> 7.4)	
TG					
Model 1	1 (Ref.)	1.01 (1.00–1.01)	1.01 (1.00–1.01)	1.01 (1.00–1.01)	< 0.05
Model 2	1 (Ref.)	1.01 (1.00–1.01)	1.01 (1.00–1.01)	1.01 (1.00–1.01)	< 0.05
Model 3	1 (Ref.)	1.01 (1.00–1.01)	1.01 (1.00–1.01)	1.01 (1.00–1.01)	< 0.05
TC					
Model 1	1 (Ref.)	1.01 (1.00–1.02)	1.01 (1.00–1.02)	1.02 (1.01–1.03)	< 0.05
Model 2	1 (Ref.)	1.01 (1.00–1.02)	1.01 (1.00–1.02)	1.02 (1.01–1.03)	< 0.05
Model 3	1 (Ref.)	1.01 (1.00–1.02)	1.01 (1.00–1.01)	1.02 (1.01–1.03)	< 0.05
HDL‐C					
Model 1	1 (Ref.)	0.94 (0.90–0.98)	0.95 (0.91–0.99)	0.93 (0.89–0.98)	< 0.05
Model 2	1 (Ref.)	0.94 (0.90–0.98)	0.95 (0.91–0.99)	0.93 (0.89–0.97)	< 0.05
Model 3	1 (Ref.)	0.93 (0.89–0.97)	0.95 (0.91–0.99)	0.93 (0.89–0.97)	< 0.05
LDL‐C					
Model 1	1 (Ref.)	1.01 (1.00–1.02)	1.01 (1.00–1.02)	1.02 (1.01–1.03)	< 0.05
Model 2	1 (Ref.)	1.01 (1.00–1.02)	1.01 (1.00–1.02)	1.02 (1.01–1.03)	< 0.05
Model 3	1 (Ref.)	1.01 (1.00–1.02)	1.01 (1.00–1.02)	1.02 (1.01–1.03)	< 0.05
TG to HDL‐C ratio					
Model 1	1 (Ref.)	1.30 (1.10–1.54)	1.34 (1.13–1.59)	1.35 (1.14–1.60)	< 0.01
Model 2	1 (Ref.)	1.32 (1.11–1.57)	1.35 (1.13–1.60)	1.36 (1.14–1.62)	< 0.01
Model 3	1 (Ref.)	1.32 (1.11–1.57)	1.34 (1.13–1.59)	1.36 (1.14–1.62)	< 0.01

*Note:* Binary logistic regression models is applied to evaluate the association between SUA and dyslipidemia components. Dependent variable is elevated dyslipidemia components defined as yes (1) or no (0), and independent variable is SUA (mg/dL). Reference category is SUA Q1. Adjusted covariates in regression model 1: age and gender; model 2: covariates in model 1 and BMI, WC and HC; model 3: covariates in model 2 and smoking and physical activity.

Abbreviations: CI, confidence interval; OR, odds ratio; SE, standard error.

## 4 Discussion

The present study evaluated the relationship between SUA levels and dyslipidemia in young adults in Bangladesh. Currently, there is limited information regarding this relationship in the young adult population. This study represents the first effort to examine the association between SUA and dyslipidemia, as well as its components, among young adults in Bangladesh.

In this study, the prevalence of dyslipidemia and its components was significantly higher in individuals with hyperuricemia than in those without hyperuricemia. We found a significant association between SUA levels and dyslipidemia after adjusting for confounders. The components of dyslipidemia, including TG, TC and LDL‐C levels, were positively associated with SUA levels, while serum HDL‐C levels were inversely related. Our results are consistent with the findings reported in Chinese [32], Polish [33], Finnish [34], Qatari [35], Brazilian [36] young adults and as well as in the adult population of Bangladesh [21], India [37], Korea [22] and the USA [20]. We also observed a positive association between the TG to HDL ratio, a well‐known marker of insulin resistance, and SUA, aligning with prior research by Keenan et al. [38]. Various clinical and epidemiological factors in these studies, and the findings indicate that hyperuricemia may lead to metabolic changes. These changes can lead to increased triglyceride (TG) levels after meals, the buildup of TG in liver tissue, and disruptions in the insulin response in the liver, adipose tissue and muscles. This understanding sheds light on the link between hyperuricemia and lipid profile indicators that we observed in our study. Notably, the significant inverse correlation between SUA and HDL‐C suggests that elevated levels may be linked to disruptions in lipid homeostasis. Therefore, SUA levels could potentially serve as a biomarker to predict the future incidence of dyslipidemia in healthy individuals.

In our study, male subjects had a higher prevalence of both hyperuricemia and dyslipidemia than female subjects, indicating a gender influence on SUA and lipid levels. Furthermore, the prevalence of dyslipidemia and its individual components was higher in the group with hyperuricemia compared to those without it. Moreover, as SUA levels increased, there was a corresponding rise in the prevalence of dyslipidemia components. A similar finding was found in other studies [32, 35, 39]. Several factors may contribute to the differences in hyperuricemia between genders. First, men typically consume higher levels of purine‐containing food and alcohol, both of which are known risk factors for hyperuricemia [40, 41]. Additionally, sex hormones may partially explain these differences. Research has shown a negative correlation between SUA levels and testosterone levels [42]. Testosterone influences the expression of urate transporters in the kidneys, including urate transporter 1, glucose transporter 9 and sodium‐coupled monocarboxylate transporter 1, which promotes uric acid reabsorption in males [43]. In the liver, testosterone also increases the activity of xanthine oxidase, the key enzyme responsible for generating uric acid [44]. Conversely, in females, the lower SUA levels may be attributed to the influence of oestrogen on post‐secretory tubular reabsorption of uric acid [45]. Therefore, it is hypothesised that the prevalence of hyperuricemia is higher in men than in women.

According to an earlier study, those with hyperuricemia have a higher risk of developing dyslipidemia than people without hyperuricemia [46]. The accumulation of visceral fat adversely affects uric acid metabolism. It results in a significant influx of free fatty acids into the liver, which stimulates TG synthesis and increases uric acid production [23, 47]. Both obesity and dyslipidemia heighten the risk of hyperuricemia, while elevated uric acid levels can also contribute to the development of obesity and dyslipidemia. It has been shown that uric acid promotes TG accumulation in cultured liver cells [48, 49]. Adopting a restricted lifestyle—by limiting alcohol, high‐purine foods and excessive fructose—can help prevent hyperuricemia. In contrast, the consumption of dairy products and fresh vegetables is encouraged. Hyperuricemia has been linked to elevated TG levels in the liver tissue of rats, likely due to oxidative stress [50]. Uric acid activates the nicotinamide adenine dinucleotide phosphate oxidase pathway, leading to endoplasmic reticulum stress and the production of reactive oxygen species in liver cells [48, 49]. Furthermore, treatment with allopurinol has been shown to effectively reduce TG, TC and fat accumulation in rats [51]. These interactions underscore the importance of considering lipid and uric metabolism in the treatment of metabolic diseases.

The role of SUA in relation to dyslipidemia and CVD remains a topic of debate. It is unclear whether elevated SUA is merely a marker of existing disorders or if it contributes causally to these conditions [20]. Research indicates that high SUA levels are a significant predictor of LDL‐C and HDL‐C particles, which are associated with an increased risk of CVD [52]. It is demonstrated that the reduction in HDL‐C promotes atherosclerosis development and increases the risk of CVD. While previous studies and biological evidence support these findings, there is a need for adequately powered prospective randomised clinical trials to establish causal relationships and to enhance clinical applications. On the other hand, the relationship between TG and uric acid levels was clear and linear [53, 54], which aligns with our findings. This relationship is thought to be influenced by genetic factors [55, 56]. It is speculated that the increased activity of the pentose phosphate pathway, which raises the demand for NADPH for fatty acid synthesis, would lead to higher NADPH production and subsequently increase uric acid production [57].

Overall, the present study emphasises a strong association between SUA levels and lipid profiles, indicating that both factors should be taken into account when considering CVD risk. Uric acid may exacerbate several mechanisms associated with CVD and interact with lipid profiles. Given the frequent coexistence of dyslipidemia and hyperuricemia, there is an urgent need for comprehensive treatment guidelines that incorporate lifestyle changes, dietary modifications and pharmacological interventions aimed at managing hyperuricemia and enhancing overall health. Furthermore, the increasing prevalence of hyperuricemia, driven by factors such as obesity and metabolic syndrome, highlights the importance of prioritising the detection and treatment of lipid and uric metabolism disorders in patients with multiple risk factors for CVD.

The current study had several limitations. First, the cross‐sectional nature of the data limited our ability to establish a causal link between SUA and dyslipidemia, highlighting the need for further research to better understand this complex relationship. Second, the sample size was relatively small, which means our findings may not be representative of the entire young adult population in Bangladesh. Therefore, future studies with larger samples are needed to confirm our results. Third, dietary habits can influence both SUA and lipid concentrations; however, we were unable to record the participants' dietary habits in this study. Fourth, medication may affect SUA and lipid profile levels; however, we did not have information about the drugs used by participants with hyperuricemia or dyslipidemia. Despite these limitations, the results of this study may serve as a valuable reference for future research.

## 5 Conclusion

The prevalence of dyslipidemia and its components was markedly higher among individuals with hyperuricemia compared to those without it. This study demonstrated a strong association between SUA levels and the prevalence of dyslipidemia and its components in young adults in Bangladesh. Further research is needed to explore the underlying mechanisms of this relationship in the younger population.

## Author Contributions

A.T. contributed to sample collection, laboratory experiments, data analysis and manuscript writing. A.D.T., S.A., J.B., F.S., and N.Z.S. assisted with sample and data collection, as well as laboratory experiments. N.A. contributed to the study concepts and design, data interpretation and writing the manuscript.

## Ethics Statement

The study protocol was approved by the Ethics Review Committee at the Department of Biochemistry and Molecular Biology, School of Life Sciences, SUST (Reference no. 02/BMB/2019).

## Conflicts of Interest

The authors declare no conflicts of interest.

## Data Availability

The data that support the findings of this study are available from the corresponding author upon reasonable request.

## References

[1] N. Schlesinger , “Dietary Factors and Hyperuricaemia,” Current Pharmaceutical Design 11 (2005): 4133–4138. 16375734 10.2174/138161205774913273

[2] R. A. Terkeltaub , “Gout,” New England Journal of Medicine 349 (2003): 1647–1655. 14573737 10.1056/NEJMcp030733

[3] L. Chen , W. Zhu , Z. Chen , et al., “Relationship Between Hyperuricemia and Metabolic Syndrome,” Journal of Zhejiang University SCIENCE B 8 (2007): 593–598. 17657863 10.1631/jzus.2007.B0593 PMC1934956

[4] A. J. Luk and P. A. Simkin , “Epidemiology of Hyperuricemia and Gout,” American Journal of Managed Care 11, no. 15 Suppl (2005): S435–S442. 16300457

[5] C. Borghi , E. A. Rosei , T. Bardin , et al., “Serum Uric Acid and the Risk of Cardiovascular and Renal Disease,” Journal of Hypertension 33 (2015): 1729–1741. 26136207 10.1097/HJH.0000000000000701

[6] N. Ali , S. Mahmood , F. Islam , et al., “Relationship Between Serum Uric Acid and Hypertension: A Cross‐Sectional Study in Bangladeshi Adults,” Scientific Reports 9 (2019): 9061. 31227765 10.1038/s41598-019-45680-4 PMC6588567

[7] N. Ali , R. Miah , M. Hasan , et al., “Association Between Serum Uric Acid and Metabolic Syndrome: A Cross‐Sectional Study in Bangladeshi Adults,” Scientific Reports 10 (2020): 1–7. 32398834 10.1038/s41598-020-64884-7 PMC7217902

[8] T. Haque , S. Rahman , S. Islam , N. H. Molla , and N. Ali , “Assessment of the Relationship Between Serum Uric Acid and Glucose Levels in Healthy, Prediabetic and Diabetic Individuals,” Diabetology and Metabolic Syndrome 11 (2019): 49. 31285758 10.1186/s13098-019-0446-6 PMC6588943

[9] N. H. Molla , R. R. Kathak , A. H. Sumon , et al., “Assessment of the Relationship Between Serum Uric Acid Levels and Liver Enzymes Activity in Bangladeshi Adults,” Scientific Reports 11 (2021): 20114. 34635716 10.1038/s41598-021-99623-z PMC8505549

[10] Z. Barman , M. Hasan , R. Miah , et al., “Association Between Hyperuricemia and Chronic Kidney Disease: A Cross‐Sectional Study in Bangladeshi Adults,” BMC Endocrine Disorders 23 (2023): 45. 36803682 10.1186/s12902-023-01304-7 PMC9942427

[11] B. Klop , J. W. F. Elte , and M. C. Cabezas , “Dyslipidemia in Obesity: Mechanisms and Potential Targets,” Nutrients 5 (2013): 1218–1240. 23584084 10.3390/nu5041218 PMC3705344

[12] N. Katsiki , D. P. Mikhailidis , and C. S. Mantzoros , “Non‐Alcoholic Fatty Liver Disease and Dyslipidemia: An Update,” Metabolism 65 (2016): 1109–1123. 27237577 10.1016/j.metabol.2016.05.003

[13] Z. Liang , Q. Y. Qiu , J. H. Wu , et al., “Alcohol Drinking, Dyslipidemia, and Diabetes: A Population‐Based Prospective Cohort Study Among Inner Mongolians in China,” Biomedical and Environmental Sciences 29 (2016): 555–562. 27660219 10.3967/bes2016.074

[14] M. Zhang , Q. Deng , L. Wang , et al., “Prevalence of Dyslipidemia and Achievement of Low‐Density Lipoprotein Cholesterol Targets in Chinese Adults: A Nationally Representative Survey of 163,641 Adults,” International Journal of Cardiology 260 (2018): 196–203. 29622441 10.1016/j.ijcard.2017.12.069

[15] M. Z. I. Chowdhury , M. A. Haque , Z. Farhana , et al., “Prevalence of Cardiovascular Disease Among Bangladeshi Adult Population: A Systematic Review and Meta‐Analysis of the Studies,” Vascular Health and Risk Management 14 (2018): 165. 30174432 10.2147/VHRM.S166111 PMC6110270

[16] A. Pirillo , M. Casula , E. Olmastroni , G. D. Norata , and A. L. Catapano , “Global Epidemiology of Dyslipidaemias,” Nature Reviews. Cardiology 18 (2021): 689–700. 33833450 10.1038/s41569-021-00541-4

[17] N. Ali , M. Samadder , R. R. Kathak , and F. Islam , “Prevalence and Factors Associated With Dyslipidemia in Bangladeshi Adults,” PLoS One 18 (2023): e0280672. 36662845 10.1371/journal.pone.0280672 PMC9857990

[18] J. P. Gonçalves , A. Oliveira , M. Severo , A. C. Santos , and C. Lopes , “Cross‐Sectional and Longitudinal Associations Between Serum Uric Acid and Metabolic Syndrome,” Endocrine 41 (2012): 450–457. 22350659 10.1007/s12020-012-9629-8

[19] M. Kuwabara , K. Niwa , I. Hisatome , et al., “Asymptomatic Hyperuricemia Without Comorbidities Predicts Cardiometabolic Diseases: Five‐Year Japanese Cohort Study,” Hypertension 69 (2017): 1036–1044. 28396536 10.1161/HYPERTENSIONAHA.116.08998 PMC5426964

[20] T.‐C. Peng , C.‐C. Wang , T.‐W. Kao , et al., “Relationship Between Hyperuricemia and Lipid Profiles in US Adults,” BioMed Research International 2015 (2015): 1–7. 10.1155/2015/127596 PMC4299312 25629033

[21] N. Ali , S. Rahman , S. Islam , et al., “The Relationship Between Serum Uric Acid and Lipid Profile in Bangladeshi Adults,” BMC Cardiovascular Disorders 19 (2019): 42. 30791868 10.1186/s12872-019-1026-2 PMC6385393

[22] M. Son , J. Seo , and S. Yang , “Association Between Dyslipidemia and Serum Uric Acid Levels in Korean Adults: Korea National Health and Nutrition Examination Survey 2016‐2017,” PLoS One 15 (2020): e0228684. 32059030 10.1371/journal.pone.0228684 PMC7021293

[23] N. Ali , R. Perveen , S. Rahman , et al., “Prevalence of Hyperuricemia and the Relationship Between Serum Uric Acid and Obesity: A Study on Bangladeshi Adults,” PLoS One 13 (2018): e0206850. 30383816 10.1371/journal.pone.0206850 PMC6211757

[24] N. Ali , N. C. Mohanto , A. Newaj , J. Begum , and F. Islam , “Evaluation of the Relationship Between Serum Uric Acid and Cardiovascular Disease: A Cross‐Sectional Study in Bangladesh,” Endocrinology, Diabetes & Metabolism 8 (2025): e70055. 10.1002/edm2.70055 PMC12045708 40312282

[25] A. Hasan , A. Newaj , A. D. Trisha , J. M. Hafsa , N. C. Mohanto , and N. Ali , “Assessment of the Relationship Between Liver Enzymes and Cardiovascular Disease: A Study in Bangladeshi Adults,” Endocrinology, Diabetes & Metabolism 7 (2024): e00481. 10.1002/edm2.481 PMC10944799 38494432

[26] N. Ali , A. Taher , N. Islam , N. Z. Sarna , and F. Islam , “Evaluation of the Relationship Between Xanthine Oxidase Activity and Metabolic Syndrome in a Population Group in Bangladesh,” Scientific Reports 14 (2024): 20380. 39223331 10.1038/s41598-024-71733-4 PMC11369145

[27] X. Sui , T. S. Church , R. A. Meriwether , F. Lobelo , and S. N. Blair , “Uric Acid and the Development of Metabolic Syndrome in Women and Men,” Metabolism ‐ Clinical and Experimental 57 (2008): 845–852. 18502269 10.1016/j.metabol.2008.01.030 PMC2486830

[28] L. You , A. Liu , G. Wuyun , H. Wu , and P. Wang , “Prevalence of Hyperuricemia and the Relationship Between Serum Uric Acid and Metabolic Syndrome in the Asian Mongolian Area,” Journal of Atherosclerosis and Thrombosis 21 (2014): 355–365. 24401703 10.5551/jat.20529

[29] Expert Panel on Detection E and Treatment of High Blood Cholesterol in Adults , “Executive Summary of the Third Report of the National Cholesterol Education Program (NCEP) Expert Panel on Detection, Evaluation, and Treatment of High Blood Cholesterol in Adults (Adult Treatment Panel III),” Journal of the American Medical Association 285 (2001): 2486–2497. 11368702 10.1001/jama.285.19.2486

[30] F. C. Bull , T. S. Maslin , and T. Armstrong , “Global Physical Activity Questionnaire (GPAQ): Nine Country Reliability and Validity Study,” Journal of Physical Activity & Health 6 (2009): 790–804. 20101923 10.1123/jpah.6.6.790

[31] N. Ali , A. H. Sumon , K. A. Fariha , et al., “Assessment of the Relationship of Serum Liver Enzymes Activity With General and Abdominal Obesity in an Urban Bangladeshi Population,” Scientific Reports 11 (2021): 1–9. 33758311 10.1038/s41598-021-86216-z PMC7988042

[32] S. Zhang , Y. Zhang , S. Lin , S. Zhang , and M. Qiu , “Hyperuricemia as a Possible Risk Factor for Abnormal Lipid Metabolism in the Chinese Population: A Cross‐Sectional Study,” Annals of Palliative Medicine 10 (2021): 11454–11463. 34872270 10.21037/apm-21-2734

[33] S. Płaczkowska , L. Pawlik‐Sobecka , I. Kokot , and A. Piwowar , “The Association Between Serum Uric Acid and Features of Metabolic Disturbances in Young Adults,” Archives of Medical Science 17 (2021): 1277–1285. 34522256 10.5114/aoms.2020.93653 PMC8425241

[34] M. Oikonen , M. Wendelin‐Saarenhovi , L.‐P. Lyytikäinen , et al., “Associations Between Serum Uric Acid and Markers of Subclinical Atherosclerosis in Young Adults. The Cardiovascular Risk in Young Finns Study,” Atherosclerosis 223 (2012): 497–503. 22749515 10.1016/j.atherosclerosis.2012.05.036

[35] Y. Al Shanableh , Y. Y. Hussein , A. H. Saidwali , et al., “Prevalence of Asymptomatic Hyperuricemia and Its Association With Prediabetes, Dyslipidemia and Subclinical Inflammation Markers Among Young Healthy Adults in Qatar,” BMC Endocrine Disorders 22 (2022): 21. 35031023 10.1186/s12902-022-00937-4 PMC8760639

[36] T. d. S. Ferreira , J. F. R. Fernandes , L. d. S. Araújo , et al., “Serum Uric Acid Levels Are Associated With Cardiometabolic Risk Factors in Healthy Young and Middle‐Aged Adults,” Arquivos Brasileiros de Cardiologia 111 (2018): 833–840. 30328946 10.5935/abc.20180197 PMC6263461

[37] D. Sarmah and B. Sharma , “A Correlative Study of Uric Acid With Lipid Profile,” Asian Journal of Medical Sciences 4 (2013): 8–14.

[38] T. Keenan , M. J. Blaha , K. Nasir , et al., “Relation of Uric Acid to Serum Levels of High‐Sensitivity C‐Reactive Protein, Triglycerides, and High‐Density Lipoprotein Cholesterol and to Hepatic Steatosis,” American Journal of Cardiology 110 (2012): 1787–1792. 22975466 10.1016/j.amjcard.2012.08.012 PMC3766845

[39] S. AlMuhaidib , F. AlBuhairan , W. Tamimi , et al., “Prevalence and Factors Associated With Dyslipidemia Among Adolescents in Saudi Arabia,” Scientific Reports 12 (2022): 16888. 36207522 10.1038/s41598-022-21262-9 PMC9547070

[40] H. K. Choi , S. Liu , and G. Curhan , “Intake of Purine‐Rich Foods, Protein, and Dairy Products and Relationship to Serum Levels of Uric Acid: The Third National Health and Nutrition Examination Survey,” Arthritis and Rheumatism 52 (2005): 283–289. 15641075 10.1002/art.20761

[41] Y. Li , Y. Jiang , M. Zhang , P. Yin , F. Wu , and W. Zhao , “Drinking Behaviour Among Men and Women in China: The 2007 China Chronic Disease and Risk Factor Surveillance,” Addiction 106 (2011): 1946–1956. 21771141 10.1111/j.1360-0443.2011.03514.x

[42] Y. Han , Y. Zhang , Y. Cao , et al., “Exploration of the Association Between Serum Uric Acid and Testosterone in Adult Males: NHANES 2011‐2016,” Translational Andrology and Urology 10 (2021): 272–282. 33532316 10.21037/tau-20-1114 PMC7844527

[43] E. Kanda , T. Muneyuki , Y. Kanno , K. Suwa , and K. Nakajima , “Uric Acid Level Has a U‐Shaped Association With Loss of Kidney Function in Healthy People: A Prospective Cohort Study,” PLoS One 10 (2015): e0118031. 25658588 10.1371/journal.pone.0118031 PMC4320097

[44] R. Hille and T. Nishino , “Flavoprotein Structure and Mechanism. 4. Xanthine Oxidase and Xanthine Dehydrogenase,” FASEB Journal 9 (1995): 995–1003. 7649415

[45] J. H. Jung , G. G. Song , Y. H. Lee , J.‐H. Kim , M. H. Hyun , and S. J. Choi , “Serum Uric Acid Levels and Hormone Therapy Type: A Retrospective Cohort Study of Postmenopausal Women,” Menopause 25 (2018): 77–81. 28796699 10.1097/GME.0000000000000953

[46] S. Chen , H. Yang , Y. Chen , et al., “Association Between Serum Uric Acid Levels and Dyslipidemia in Chinese Adults: A Cross‐Sectional Study and Further Meta‐Analysis,” Medicine 99 (2020): e19088. 32176036 10.1097/MD.0000000000019088 PMC7440131

[47] I. Fabregat , E. Revilla , and A. Machado , “Short‐Term Control of the Pentose Phosphate Cycle by Insulin Could Be Modulated by the NADPHNADP Ratio in Rat Adipocytes and Hepatocytes,” Biochemical and Biophysical Research Communications 146 (1987): 920–925. 3304289 10.1016/0006-291x(87)90618-8

[48] M. A. Lanaspa , L. G. Sanchez‐Lozada , Y.‐J. Choi , et al., “Uric Acid Induces Hepatic Steatosis by Generation of Mitochondrial Oxidative Stress: Potential Role in Fructose‐Dependent and Independent Fatty Liver,” Journal of Biological Chemistry 287 (2012): 40732–40744. 23035112 10.1074/jbc.M112.399899 PMC3504786

[49] Y.‐J. Choi , H.‐S. Shin , H. S. Choi , et al., “Uric Acid Induces Fat Accumulation via Generation of Endoplasmic Reticulum Stress and SREBP‐1c Activation in Hepatocytes,” Laboratory Investigation 94 (2014): 1114–1125. 25111690 10.1038/labinvest.2014.98

[50] E. Tapia , M. Cristóbal , F. E. García‐Arroyo , et al., “Synergistic Effect of Uricase Blockade Plus Physiological Amounts of Fructose‐Glucose on Glomerular Hypertension and Oxidative Stress in Rats,” American Journal of Physiology. Renal Physiology 304 (2013): F727–F736. 23303409 10.1152/ajprenal.00485.2012 PMC3602695

[51] W. Wang , C. Wang , X.‐Q. Ding , et al., “Quercetin and Allopurinol Reduce Liver Thioredoxin‐Interacting Protein to Alleviate Inflammation and Lipid Accumulation in Diabetic Rats,” British Journal of Pharmacology 169 (2013): 1352–1371. 23647015 10.1111/bph.12226 PMC3831713

[52] J. Vekic , Z. Jelic‐Ivanovic , V. Spasojevic‐Kalimanovska , et al., “High Serum Uric Acid and Low‐Grade Inflammation Are Associated With Smaller LDL and HDL Particles,” Atherosclerosis 203 (2009): 236–242. 18603253 10.1016/j.atherosclerosis.2008.05.047

[53] D. Conen , V. Wietlisbach , P. Bovet , et al., “Prevalence of Hyperuricemia and Relation of Serum Uric Acid With Cardiovascular Risk Factors in a Developing Country,” BMC Public Health 4 (2004): 9. 15043756 10.1186/1471-2458-4-9 PMC406506

[54] J. T. Moriarity , A. R. Folsom , C. Iribarren , F. J. Nieto , and W. D. Rosamond , “Serum Uric Acid and Risk of Coronary Heart Disease: Atherosclerosis Risk in Communities (ARIC) Study,” Annals of Epidemiology 10 (2000): 136–143. 10813506 10.1016/s1047-2797(99)00037-x

[55] G. A. A. Ferns , J. Lanham , P. Dieppe , and D. J. Galton , “A DNA Polymorphism of an Apoprotein Gene Associates With the Hypertriglyceridaemia of Primary Gout,” Human Genetics 78 (1988): 55–59. 2892776 10.1007/BF00291235

[56] Y. Moriwaki , T. Yamamoto , S. Takahashi , Z. Tsutsumi , and K. Higashino , “Apolipoprotein E Phenotypes in Patients With Gout: Relation With Hypertriglyceridaemia,” Annals of the Rheumatic Diseases 54 (1995): 351–354. 7794039 10.1136/ard.54.5.351 PMC1005593

[57] H. Vuorinen‐Markkola and H. Yki‐Järvinen , “Hyperuricemia and Insulin Resistance,” Journal of Clinical Endocrinology & Metabolism 78 (1994): 25–29. 8288709 10.1210/jcem.78.1.8288709

